# Campylobacter fetus Cellulitis

**DOI:** 10.7759/cureus.35328

**Published:** 2023-02-22

**Authors:** Lia Bastos, Ricardo Gomes, Sara Pocinho, Teresa Baptista, Kamal Mansinho

**Affiliations:** 1 Infectious Diseases and Tropical Medicine, Centro Hospitalar de Lisboa Ocidental, Lisbon, PRT; 2 Internal Medicine, Centro Hospitalar Universitário Cova da Beira, Covilhã, PRT

**Keywords:** bacteremia, immune suppression, portugal, cellulitis, campylobacter fetus

## Abstract

*Campylobacter fetus*, a bacteria of the *Campylobacter* genus that are a group of bacteria known to cause intestinal infections, is a particular microbial agent due to its most common presentation being as a non-intestinal systemic infection and rarely as a focal infection, most frequently cellulitis. *C. fetus*’s main reservoirs are cattle and sheep. Humans are usually infected by consuming raw milk and/or meat. Infection in humans is rare and generally related to immune deficiencies, malignancy, chronic liver disease, diabetes mellitus and elderly age, among other factors. Diagnosis is usually achieved by blood cultures due to the lack of focalized signs/symptoms and the pathogen’s endovascular tropism. The authors present a case of cellulitis due to *Campylobacter fetus*, a microbial agent that affects susceptible patients with a mortality rate of up to 14%. We aim to emphasize the importance of potential bacterial seeding sites secondary to bacteremia given the agent tropism for vascular tissue. The medical diagnosis was performed by the identification of bacteria in blood cultures. *Campylobacter spp.* infections are more frequently related to undercooked poultry or meat, but in this case, the consumption of fresh cheese was considered the most likely source of infection. A literature review showed that, in patients with previous antibiotic cycles, a combination of carbapenem and gentamicin had better outcomes and lower relapse rates. Due to typical surface antigenic variation, immune control may not be attainable and may account for relapsing infections, even after appropriate therapy. The duration of treatment has yet to be well established. Based on other reported cases, we considered a four-week treatment to be sufficient, given clinical improvement and absence of recurrence in the follow-up time.

## Introduction

*Campylobacter fetus *is the third most common species of bacteria of the* Campylobacter genus. Campylobacter spp. *are recognized as the leading cause of bacterial enteric infections worldwide, but* Campylobacter fetus* is rarely isolated on stools (<1%) as it more frequently causes systemic infection and is related to higher rates of hospitalization [1]. *C. fetus*'s main reservoirs are cattle and sheep, and humans are usually infected by consuming raw meat and/or milk [2].

*C. fetus* infections are rare and occur in patients presenting with underlying immune deficiencies, malignancy, diabetes mellitus, chronic liver disease, elderly age or under immunosuppressive treatment [3]. They cause bacteremia which may be related or not to secondary localizations. When metastatic infection is present, the most common localizations are the vascular system (aneurysms or vascular prosthesis infections) or cutaneous [3]. The protein surface layer of this microorganism allows it to evade opsonization, leading to easier invasion of extraintestinal infectious focus with particular tropism for the human vascular endothelium due to bacterial surface receptors [4]. Diagnosis is usually achieved by blood cultures due to the lack of focalized signs/symptoms and the pathogen’s endovascular tropism [3].

Invasive campylobacter diseases have no clear treatment guidelines. There are conflicting reports on the therapeutic usefulness and failure of third-generation cephalosporins as an empiric monotherapy. A previous experience by specialists supported the use of gentamicin as empiric therapy for invasive campylobacteriosis given that it has a low minimum inhibitory concentration (MIC) and there is rare gentamicin resistance reported in the studies to date. Carbapenems including imipenem are considered a wise empiric choice in monotherapy because *C. fetus *has sustained sensitivity reports to them and reported favorable outcomes were documented [4].

## Case presentation

An 81-year-old male living in an urban environment spent his weekends in a country house where he usually consumed fresh cheese and was evaluated in the hematooncology consultation complaining of pain, rubor and edema in the left leg that appeared suddenly and without trauma. After medical evaluation and the diagnosis of cellulitis, he was treated in ambulatory with flucloxacillin 2 g daily orally for 14 days. He presented with ischemic cardiomyopathy, diabetes mellitus, peripheral artery disease and monoclonal gammopathy of undetermined significance as underlying medical conditions, and due to a recently diagnosed non-Hodgkin B-cell lymphoma, he was on a regimen with prednisolone 30 mg on alternated days for two months and completed two cycles of rituximab in the previous two months.

Without clinical improvement after antimicrobial therapy and maintaining elevated inflammatory parameters, ambulatory blood cultures were collected that revealed *C. fetus*. The patient was then admitted to the hospital and started on ertapenem and gentamicin intravenously and completed a total of 14 days of ertapenem and seven days of gentamicin combined therapy in the second week with clinical improvement in a hospital-at-home setting. The intravenous therapy was administered by peripheral venous catheter, and the patient received medical care at home allowing for a more personal and caring environment. The cellulitis was treated, and the patient was discharged.

Two weeks later, he went to the emergency department because of a recurrence of pain, rubor and edema on his left leg diagnosed as cellulitis. Blood work showed anemia (hemoglobin (Hg) 7.8 g/dL), thrombocytopenia (platelets 87,000) and elevated C-reactive protein (22.2 mg/dL). The patient was admitted to the hospital, empiric therapy with amoxicillin/clavulanate 1,200 mg thrice a day was initiated and the patient was transferred to the Infectious Diseases ward.

**Figure 1:**
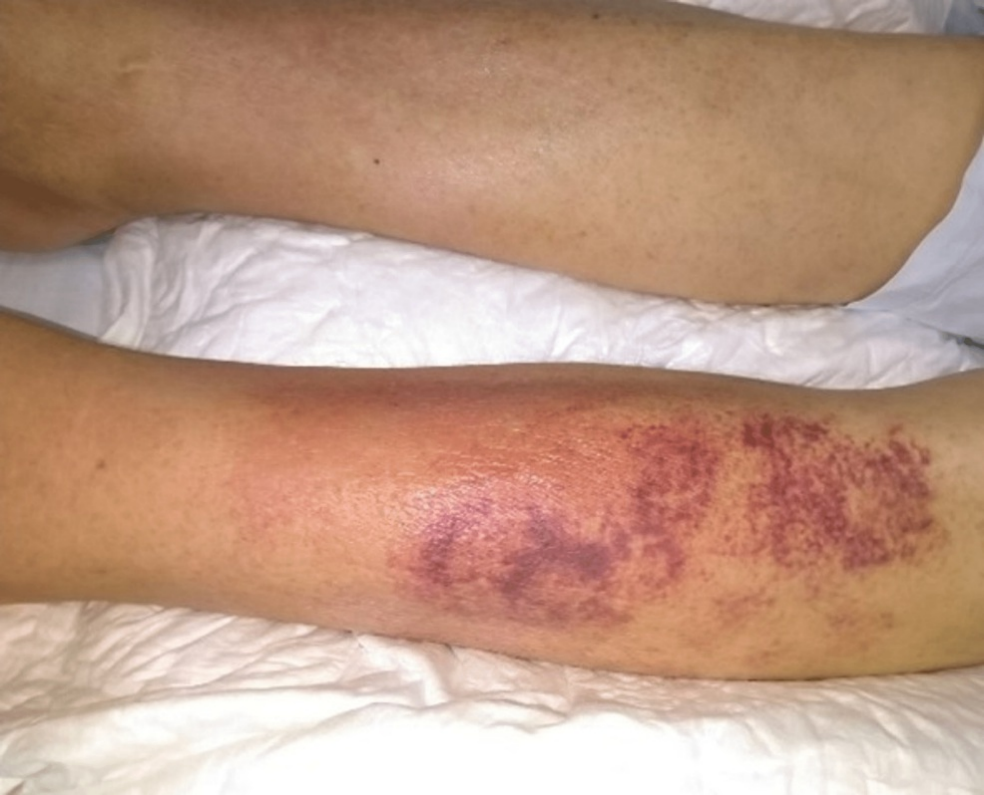
Cellulitis on admission

**Figure 2:**
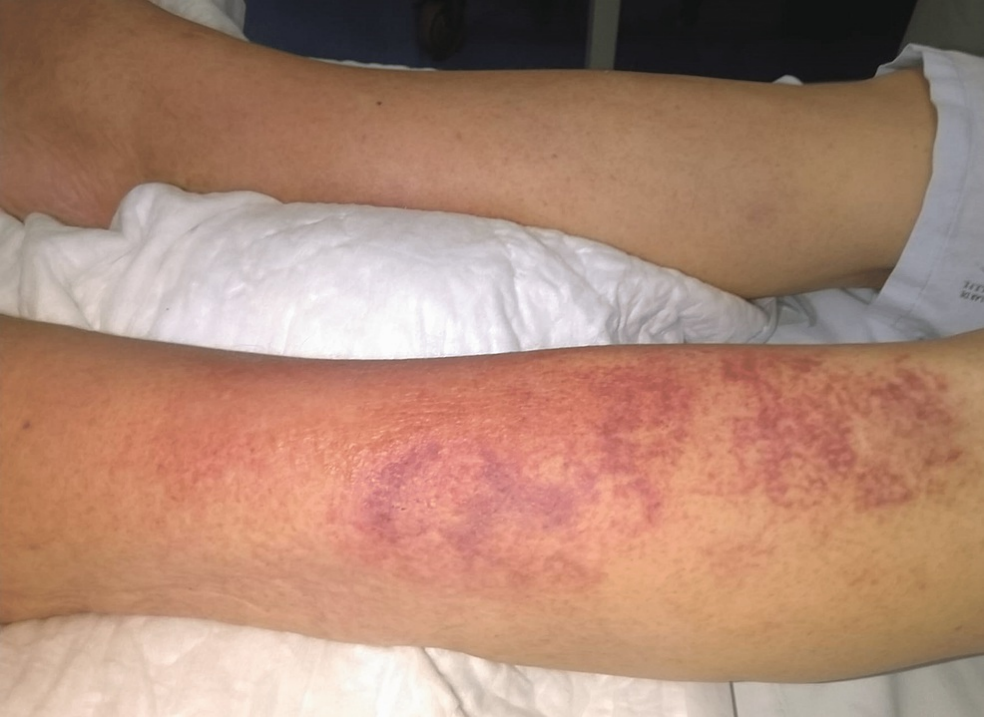
Cellulitis after two weeks on meropenem

**Figure 3:**
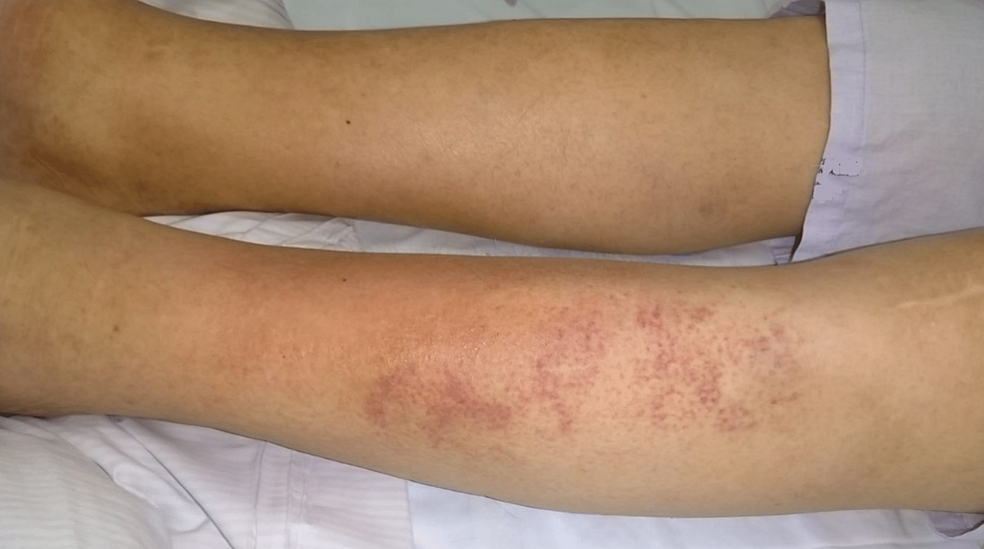
Cellulitis after three weeks of meropenem and one week of gentamicin (combined therapy)

Other than cellulitis, the patient presented with acute heart failure (peripheral edemas and asthenia) and aggravated cytopenia, and new positive blood cultures were collected isolating *C. fetus* (sensible to ciprofloxacin and doxycycline and resistant to erythromycin). Stool cultures were negative. He was started on meropenem to which, after one week, gentamicin was added and completed a total of four weeks of meropenem and three weeks of gentamicin. Diuretic therapy and transfusion of 2 units of erythrocytes were performed, and the patient improved significantly solving the peripheral edema and asthenia. Prednisolone (previously prescribed by hematooncology as a treatment for non-Hodgkin lymphoma) was suspended until further evaluation by this medical specialty. Improved clinical response was observed as documented in Figures 1-3.

Given this agent tropism for endovascular territory, we performed lower limbs Doppler echography; angiography of the lower limbs, abdomen and chest; and positron emission tomography (PET) scan which excluded disseminated disease or localized abscesses. The patient was discharged and returned home after complete treatment in the hospital ward. At the six-, 12- and 18-month follow-ups, the patient showed no relapsing disease.

## Discussion

The clinical presentation of *C. fetus* infection in humans may vary from an acute diarrheal episode to a systemic illness whose signs and symptoms are related to the localization where the bacteria has spread as an example of metastatic infection [5]. Sepsis with fever without apparent localized infection is reported in 24% up to 41% of cases. Focal infection compromising the central nervous system, osteomyelitis, lung abscesses, arthritis and perinatal infections can also occur [6]. Vascular infection (mycotic aneurysms, vasculitis) poses specific risks due to bacteria seeding properties and a greater risk of thromboembolic septic events (thrombophlebitis, endocarditis and pericarditis). Cellulitis is a clinical entity that is more frequently the source of bacteremia from cutaneous origin, but in *C. fetus*,* *infection is the principal extraintestinal manifestation of bacteremia [7].

The patient presented risk factors for severe disease such as age, hematological malignancy, diabetes mellitus and iatrogenic immunosuppression, mounting an insufficient immune response and a greater risk of relapsing. Even though the patient lived in an urban environment, he spent his weekends in a country house where he usually consumed fresh cheese. He denied any contact with animals, including cattle and sheep. He has had a dog 10 years before but currently did not have any pets. Given the dietary habits of the patient, clinical history and epidemiological context, we considered the oral ingestion the most probable route of infection [8].

Adaptive mechanisms of this agent, whose surface layers of the cell walls form a capsule-like structure, comprising an array of S-layer proteins which confer resistance to the complement-mediated killing of the bacteria, in association with non-Hodgkin lymphoma, rituximab and corticosteroid therapy (severe immunosuppression), may have contributed to infection persistence and clinical relapse [8]. Diagnosis of this entity remains challenging as clinical manifestations vary greatly and bacterial identification is necessary. *C. fetus* are fastidious microorganisms requiring specific microaerobic growth conditions [6].

There is no consensus on treatment choice or duration, but clinical experience sustained the importance of antibiotic therapy for immunocompromised and geriatric patients, with a higher death rate in patients without appropriate antibiotic treatment [9]. Empirical treatment with fluoroquinolones of *C. fetus* bacteremia was associated with higher resistance frequency with fatal outcomes. Gentamicin has very low MICs for *C. fetus*, and no gentamicin-resistant *C. fetus* strains were found in a contemporary study from Quebec [9]. Sustained by clinical experience and microbiological data, many experts support regimens that include gentamicin to treat severe Campylobacter bacteremia and endovascular infection. Most *C. fetus* strains are resistant to penicillins due to beta-lactamases properties and its response to different beta-lactam is quite erratic; due to the lack of strain identification of the bacteria, it is prudent to use carbapenems whose resistance is rare [9].

## Conclusions

*Campylobacter fetus* is an opportunistic organism which usually infects immunocompromised patients. In this case, the consumption of fresh cheese was considered the most likely source of infection. As reported in the literature, carbapenem on monotherapy has good clinical outcomes, but given the severe immunocompromised state and recurrent infection on a patient who had already completed ertapenem and gentamicin, we opted for a combination of meropenem with gentamicin with favorable clinical outcome. The treatment regimen duration specific for this illness has not been defined, but previous studies revealed that at least three weeks is needed to avoid clinical relapse, so we considered four weeks of treatment to be sufficient, given the clinical improvement and the absence of relapse in the time of follow-up (more than one year). This clinical case presents a rare clinical entity and reminds clinicians to be aware of the specificities of an uncommon pathogen, especially in vulnerable patients.
